# SIRT3 Overexpression Attenuates Palmitate-Induced Pancreatic β-Cell Dysfunction

**DOI:** 10.1371/journal.pone.0124744

**Published:** 2015-04-27

**Authors:** Min Kim, Ji Seon Lee, Joo En Oh, Jinyan Nan, Hakmo Lee, Hye Seung Jung, Sung Soo Chung, Kyong Soo Park

**Affiliations:** 1 Department of Internal Medicine, Seoul National University College of Medicine, Seoul, Korea; 2 Department of Molecular Medicine and Biopharmaceutical Sciences, Graduate School of Convergence Science and Technology, College of Medicine, Seoul National University, Seoul, Korea; CRCHUM-Montreal Diabetes Research Center, CANADA

## Abstract

Abnormally high levels of circulating free fatty acids can lead to pancreatic islet β-cell dysfunction and apoptosis, contributing to β-cell failure in Type 2 diabetes. The NAD^+^-dependent protein deacetylase Sirtuin-3 (SIRT3) has been implicated in Type 2 diabetes. In this study, we tested whether SIRT3 overexpression affects palmitate-induced β-cell dysfunction in cells of line NIT1, which are derived from mouse pancreatic β-cells. Two different lengths of SIRT3 were overexpressed: full length SIRT3 (SIRT3LF), which was preferentially targeted to mitochondria and partially to the nucleus, and its N-terminal truncated form (SIRT3SF), which was located in the nucleus and cytoplasm. Overexpression of SIRT3LF and SIRT3SF using an adenoviral system alleviated palmitate-induced lipotoxicity such as reduction of cell viability and mitogen-activated protein kinase (MAPK) activation. Chronic exposure to low concentrations of palmitate suppressed glucose-stimulated insulin secretion, but the suppression was effectively reversed by overexpression of SIRT3LF or SIRT3SF. The mRNA levels of the endoplasmic reticulum (ER) stress responsive genes ATF4, GRP94 and FKBP11 were increased by palmitate treatment, but the increases were completely inhibited by SIRT3LF overexpression and less effectively inhibited by SIRT3SF overexpression. This result suggests that overexpression of SIRT3 inhibits induction of ER stress by palmitate. Collectively, we conclude that overexpression of SIRT3 alleviates palmitate-induced β-cell dysfunction.

## Introduction

The NAD^+^-dependent protein deacetylase Sirtuin-3 (SIRT3) is a member of the sirtuin family of proteins [1,2]. SIRT3 contains mitochondrial localization sequences that direct its import into the mitochondria, where it is cleaved to a shorter form [3,4]. Many proteins of mitochondrial metabolic pathways, such as the tricarboxylic acid pathway, oxidative phosphorylation, and fatty acid β-oxidation, are regulated by acetylation [5]. The importance of the deacetylation activity of SIRT3 in mitochondria is evidenced by the demonstration of hyperacetylation of mitochondrial proteins in SIRT3^-/-^ mice [6]. In response to fasting, SIRT3 expression increased in the liver accompanied by altered fatty acid metabolism, whereas mice fed a high-fat diet had lower SIRT3 expression and activity in liver and skeletal muscle [7]. Also SIRT3-deficient mice were more likely than normal mice to develop insulin resistance and obesity [8–10].

Only a little has been reported for the role of SIRT3 in pancreatic β cells, and the evidence supporting the importance of SIRT3 in pancreatic islet β cells related to Type 2 diabetes can be summarized as follows. SIRT3 levels are lower in pancreatic islets in human patients afflicted with Type 2 diabetes. When cells of the INS1 line, which are derived from rat pancreatic β cells, were treated with interleukin-1β (IL1β) or tumor necrosis factor α (TNFα), SIRT3 levels declined compared to untreated cells [11]. High passage MIN6 cells, a mouse pancreatic β-cell line, had reduced SIRT3 expression [12]. Knock-down of SIRT3 in INS1 cells decreased insulin secretion and increased the levels of reactive oxygen species (ROS) and apoptosis compared to the wild type. Finally, it has been reported that the protective effects of nicotinamide mononucleotide against TNFα or IL1β treatment are mediated by SIRT3 [11].

The viability and insulin secretion of pancreatic β cells are reduced by high-fat conditions, especially by high levels of palmitate [13–15]. Although the molecular mechanism underlying lipotoxicity is not fully understood, ROS has been considered to be an important factor mediating lipotoxicity in islet β cells [13]. Expression of antioxidant genes is quite low in islet β cells, making them labile to oxidative stress [16,17]. ROS is related to protein misfolding in the endoplasmic reticulum (ER) and induces ER stress. Several reports have shown that palmitate induces ER stress and leads to β-cell dysfunction and apoptosis [18–20]. There have been various attempts to protect β cells from lipotoxicity [14,21]. For example, ROS inhibition by antioxidants ameliorated palmitate-induced ER stress and cell death in INS1 cells [18]. Activation of SIRT1 counteracted the inhibition by palmitate of insulin transcription [22].

In this study, we asked if SIRT3 overexpression could protect islet β cells from the negative effects of palmitate. It is generally accepted that SIRT3 contains a mitochondria import signal and is cleaved to a shorter active form in mitochondria [2–4]. However, there is some evidence that SIRT3 is also found in the nucleus [23,24]. The relationship between localization of SIRT3 and its function was examined by using two forms of SIRT3, a full-sized SIRT3 and an N-terminal truncated form. The mitochondria localization signal should be present in the former and absent in the truncated form.

## Materials and Methods

### Cell culture and palmitate treatment

NIT1, a pancreatic beta-cell line established from a transgenic NOD/Lt mouse, was purchased from ATCC (NO. CRL-2055, Manassas, VA, USA) and cultured in RPMI 1640 medium (Gibco Life Technologies, Carlsbad, CA, USA) supplemented with 10% fetal bovine serum (FBS, Gibco Life Technologies), 100 U/mL penicillin, and 0.1 mg/mL streptomycin. Palmitate was dissolved at 100 mM in ethanol to make stock solution. Palmitate stock solution was diluted in the culture medium to which fatty-acid-free BSA had been added, in a 1:3 molar ratio, to prepare BSA-conjugated palmitate. Cells were incubated with the BSA-conjugated-palmitate (50–500 μM) for the indicated time periods.

### Plasmids, adenovirus, and antibodies

An expression vector for the long form of human SIRT3 (pcDNA-SIRT3LF) was constructed by sub-cloning the full length of SIRT3 cDNA fragment into a pcDNA3.1 vector (Invitrogen, Carlsbad, CA, USA). The expression vector for the short form of human SIRT3 (pcDNA-SIRT3SF) was constructed by subcloning a SIRT3 cDNA fragment lacking the N-terminal 141 amino acids into a pcDNA3.1 vector (Invitrogen). Adenoviruses overexpressing the long and short forms (Ad-SIRT3LF and Ad-SIRT3SF, respectively) were generated: Both forms of SIRT3 cDNA were inserted into the pAdTrack-CMV vector and pShuttle-CMV vector, followed by recombination with pAdEasy adenoviral backbone vectors. The pAd-Track-CMV vector, but not pShuttle-CMV vector, contains GFP expression system. Adenoviruses were generated by transfecting the recombinant adenoviral DNA into 293 cells and purified by CsCl density gradient centrifugation. For SIRT3 localization experiments, pShuttle-CMV generated adenovirus was used to avoid GFP expression and Ad-β-gal was used as control. For other experiments, pAdTrack-CMV generated adenovirus was used and infection rate was determined by green fluorescence by GFP. More than 80% cells were infected in each experiment. Antibodies against p-p38, p-Erk1/2, p38, Erk1/2 and acetylated lysine (Ac-Lys) were purchased from Cell Signaling Technology (Danvers, MA, USA). Antibodies against poly (ADP-ribose) polymerase (PARP) and enolase were purchased from Santa Cruz Biotechnology (Santa Cruz, CA, USA). Prohibitin monoclonal antibody was purchased from Neomarkers (Fremont, CA, USA) and antibodies against γ-tubulin and SIRT3 were purchased from Sigma-Aldrich (St. Louis, MO, USA).

### Caspase 3 activity assay

Cells were seeded in a 12-well plate and infected with the adenoviruses (50 MOI) for 24 h then treated with 500 μM palmitate for 24 h. Caspase-3 activity was measured by using Caspase-3/CPP32 activity Colorimetric Assay Kit (BioVision, Milpitas, CA, USA). Cells were lysed with lysis buffer supplied in the kit and incubated on ice for 10 min, and centrifuged at 10,000 x g for 5 min. The supernatant was collected and protein concentration was determined. 100 μg of protein was diluted to 50 μl with lysis buffer for each assay. The lysate was mixed with 50 μl of 2X reaction buffer containing 10 mM DTT and 5 μl of the 4 mM DEVD-pNA substrate was added. After incubation at 37°C for 2 h, samples were read at 405 nm using a microplate reader.

### Measurement of cellular ATP levels

Cellular ATP levels were determined using the ATPLite kit (PerkinElmer life Science, Boston, MA, USA). Cells were seeded in a 24-well plate and infected with the adenoviruses (50 MOI) for 24 h and treated with palmitate for 8 h. Cells were lysed with cell lysis solution that had been supplied in the kit and 50 μL of the lysate was transferred to a 96-well plate. Fifty microliters of the substrate solution, also supplied, was added to each well. The luminescence was measured using a Victor 3 1420 multilabel counter (PerkinElmer, Boston, MA, USA).

### Measurement of glucose-stimulated insulin secretion

NIT1 cells were treated with adenovirus for 24 h in RPMI 1640 supplemented with 10% FBS, after which 200 μM palmitate was added to the medium for 48 h. Cells were starved for 2 h in glucose-deficient RPMI 1640. Cells were washed twice with a glucose-free Krebs-Ringer-HEPES buffer (KRH; 119 mM NaCl, 4.74 mM KCl, 2.54 mM CaCl_2_, 1.19 mM KH_2_PO_4_, 1.19 mM MgCl_2_, 10 mM HEPES, 25mM NaHCO_3_, 0.2% BSA). The cells were incubated in glucose-free KRH buffer for 1h and then the medium was replaced with KRH containing 2.5 mM (low glucose) or 16.7 mM (high glucose) glucose. After 1 hour exposure to the low or high concentration of glucose, the supernatants were collected to measure insulin level. Insulin was measured by the insulin immunoassay kit (ALPCO, Salem, NH, USA) according to the manufacturer’s instructions.

### Animal care and isolation of rat pancreatic islets and analyses of insulin secretion

All aspects of animal care and experimentation were conducted in accordance with the Guide for the Care and Use of Laboratory Animals of the National Institutes of Health and approved by the Institutional Animal Care and Use Committees of Seoul National University Hospital (Permit Number: SNU-111110-3-2). All surgery was performed under anesthesia with zoletil and xylazine, and all efforts were made to minimize suffering.

Isolation of islets from rat was carried out according to the protocol previously described [25]. In brief, after clamping the common bile duct, Hank’s buffered salt solution (HBSS) containing 0.8 mg/ml collagenase was injected into the duct. The swollen pancreas was removed and digested in HBSS containing 0.8 mg/ml collagenase at 37°C for 15min. To terminate digestion, HBSS containing 10% FBS and 10 mM HEPES was added. Then the solution was filtered with 500 μm mesh and islets were collected by centrifugation at 1500 rpm for 2 min. Islets were purified via ficoll gradient centrifugation and the 100–200 μm size islets were picked. Selected islets were incubated at 37°C in culture media (RPMI 1640 supplemented with 10% FBS, 100 U/mL penicillin, and 0.1 mg/mL streptomycin) for 24 h. Islets were infected with adenoviruses (100 MOI) for 24 h and 400 μM palmitate was treated for additional 48 h. After incubating for 2 h in low glucose KRH buffer, islets were incubated with fresh low glucose KRH. After 30 min exposure, the buffer was collected and islets were incubated with high glucose KRH buffer for 30 min. Insulin in the buffer was measured by the insulin immunoassay kit (ALPCO, Salem, NH, USA) according to the manufacturer’s instructions.

### Subcellular fractionation

NIT1 cells were seeded in 100 mm dishes and infected with Ad-SIRT3LF (50 MOI), Ad-SIRT3SF (200 MOI) or Ad-β-gal (control, 200 MOI) for 48 h. Cells were collected, washed once with PBS and suspended in the mitochondria isolation buffer (MIB; 0.1 mM EDTA, 10 mM Tris—HCl, 250 mM Sucrose) supplemented with 1 mM PMSF, 7 μg/mL leupeptin, and 7 μg/mL aprotinin. Cell suspensions were then homogenized with 40 strokes in a glass-teflon homogenizer on ice and centrifuged at 1,000 x g for 15 min at 4°C. The pellets (nucleus) were collected and the supernatants were centrifuged at 10,000 x g for 30 min at 4°C. The supernatant was kept as the cytosolic fraction and the pellets (mitochondria) were collected. The pellets containing nuclear fraction or mitochondrial fraction were washed with MIB twice and suspended with the lysis buffer for western blot analysis.

### Western blot analysis

Cell lysates were prepared in the lysis buffer containing 20 mM Tris—HCl (pH 7.4), 1% NP-40, 10 mM Na_4_P_2_O_7_, 5 mM EDTA, 100 mM NaF, 2 mM Na_3_VO_4_, 7 μg/mL leupeptin, 7 μg/mL aprotinin, and 1 mM phenylmethylsulfonyl fluoride. Whole-cell lysates were subjected to sodium dodecyl sulfate—polyacrylamide gel electrophoresis (SDS-PAGE). Proteins on the gel were transferred onto a nitrocellulose membrane. Membranes were incubated with blocking solution (5% skim milk), and then incubated with specific primary antibodies in 0.1% Tween 20-Tris-buffered saline. Hybridized primary antibodies were detected using a horseradish peroxidase-conjugated IgG antibody (Santa Cruz Biotechnology). Bands were detected by using the enhanced chemiluminescence kit (Thermo, Rockford, IL, USA).

### Immunocytochemistry and confocal image

NIT1 cells were seeded in a 12-well plate and infected with Ad-SIRT3LF (50 MOI) or Ad-SIRT3SF (200 MOI) and Ad-β-gal (control, 200 MOI) for 48 h. The cells were transferred to chamber slides for 24 h, after which they were incubated with 500 nM MitoTracker Deep red FM (Invitrogen) in growth medium at 37°C for 45 min. Cells were rinsed with phosphate-buffered saline (PBS), fixed for 5min with 3.7% formaldehyde in PBS, and blocked with PBS-Tween 20 (PBS-T, 0.05%) supplemented with 5% normal goat serum (NGS) for 1 h. Cells were incubated with monoclonal antibody of SIRT3 (1:100) in 2.5% NGS at 4°C overnight, washed with PBS-T three times for 5 min, and incubated with goat anti-rabbit Alexa 488 (Invitrogen) at a 1:500 dilution in 2.5% NGS for 1 h. After washing with PBS-T three times for 5 min and staining with 1 μM 4’,6-diamidino-2-phenyldole (DAPI) for 30 s, the slides were mounted and sealed for analysis in a confocal microscope (Leica Microsystems, Wetzlar, Germany) fitted with the appropriate filters.

### RNA preparation and real-time quantitative PCR

Total RNA was extracted using TRIzol reagent (Invitrogen) according to the manufacturer’s instructions. The cDNAs were prepared by reverse transcription with 1 μg of total RNA. Real-time PCR was performed using SYBR Premix Ex Taq reagents (TaKaRa, Shiga, Japan) and a 7500 real-time PCR system (Applied Biosystems, CA, USA). The glyceraldehyde 3-phosphate dehydrogenase (GAPDH) mRNA level was used for the internal control. Experiments were performed in duplicate for each sample. Primer sequences of ATF4 were: 5’-CCTGAACAGCGAAGTGTTGG-3’ (forward), 5’- TGGAGAACCCATGAGGTTTCAA-3’ (reverse), GRP94: 5’- AAACGGCAACACTTCGGTCAG -3’ (forward), 5’- GCATCCATCTCTTCTCCCTCATC -3’ (reverse), FKBP11: 5’-ACACGCTCCACATACACTACACGG-3’ (forward), 5’- ATGACTGCTCTT CGCTTCTCTCCC -3’ (reverse). IL1β: 5’-TGCAGAGTTCCCCAACTGGTACATC-3’ (forward), 5’-GTGCTGCCCTAATGTCCCCTTGAATC-3’ (reverse), IL6: 5’- GATGCTGGTGACAACCACGG -3’ (forward), 5’- TTCTCATTTCCACGATTTCCCA -3’ (reverse), TXNIP: 5’-TATGTACGCCCCTGAGTTCC-3’ (forward), 5’-GCTCACTGCACGTTGTTGTT-3’ (reverse), SIRT3: 5’-GCTGCTTCTGCGGCTCTATAC-3’ (forward), 5’-GAAGGACCTTCGACAGACCGT-3’ (reverse).

### Statistical analysis

Statistical analysis was performed using SPSS version 12.0 (SPSS Inc.). Statistical significance was tested using the Mann-Whitney U test. A *P* value below 0.05 was considered statistically significant.

## Results

### Overexpression of SIRT3 and cellular localization

**Fig 1:**
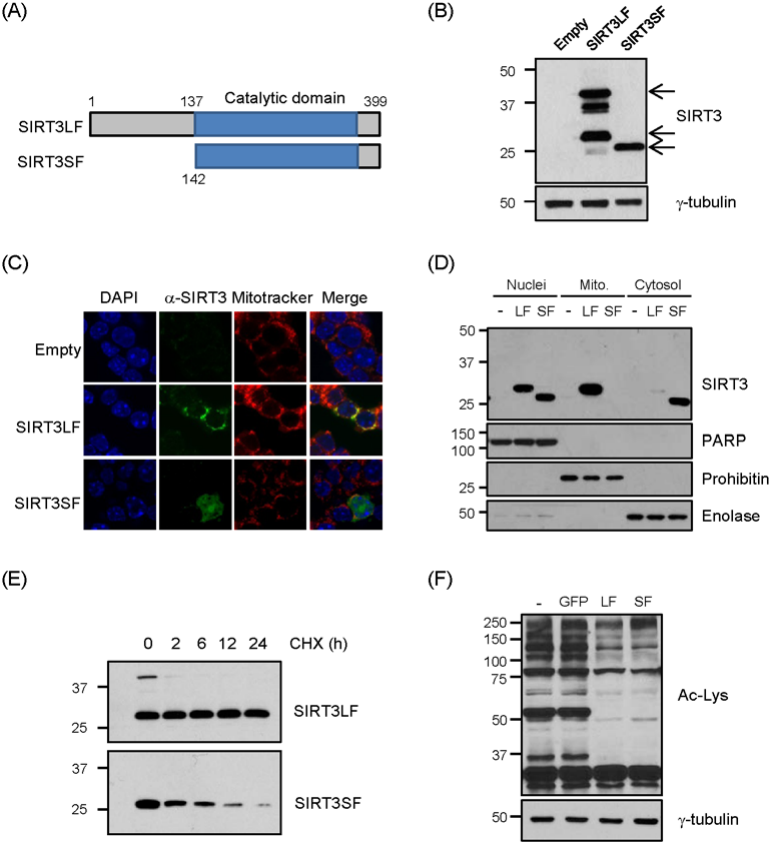
Localization of SIRT3LF and SIRT3SF. (A) Expression vectors and adenovirus expressing full-length human SIRT3 (SIRT3LF) and N-terminal region-truncated SIRT3 (SIRT3SF, with 141 N-terminal amino acids deleted) were generated. (B) NIT1 cells were infected with Ad-SIRT3LF (50 MOI), Ad-SIRT3SF (200 MOI) and Ad-β-gal (Empty, 200 MOI). Ten μg proteins were loaded on each lane and immunoblotted with a SIRT3 antibody. (C) NIT1 cells were infected with Ad-β-gal (Empty), Ad-SIRT3LF or Ad-SIRT3SF for 48 h. SIRT3 (green) was detected by immunofluorescence confocal microscopy. Mitochondria (red) were stained with Mitotracker, and nuclei were stained with DAPI (blue). (D) NIT1 cells were infected with Ad-SIRT3LF or Ad-SIRT3SF for 48h, and then subjected to subcellular fractionation. 15 μg proteins were used for Western blot analyses. Subcellular fractionation was confirmed by immunoblot with antibodies against PARP (nucleus), prohibitin (mitochondria) and enolase (cytosol). (E) COS7 cells were transfected with pcDNA-SIRT3LF (100 ng) or pcDNA-SIRT3SF (400 ng), and 24 h later the cells were treated with cycloheximide (CHX, 5 μM). Cell lysates were prepared after the CHX treatment, followed by Western blot analysis with anti-SIRT3 antibody. (F) NIT1 cells were infected with Ad-GFP (empty), Ad-SIRT3LF or Ad-SIRT3SF (all 50 MOI) for 48 h and trichostatin A (1 μM) was added 6 h before harvest. Whole cell lysates were subjected to Western blot analyses with anti-acetylated lysine (Ac-Lys) antibody.

Expression vectors were generated for two lengths of human SIRT3: the long form (SIRT3LF) and the N-terminal truncated short form (SIRT3SF; Fig 1A). Two different lengths of SIRT3 were overexpressed in NIT1 cells using the adenoviral system (Ad-SIRT3LF and Ad-SIRT3SF). Two major different lengths of SIRT3 were detected in NIT1 cells infected with Ad-SIRT3LF in the Western blot analysis. In contrast, a single, smaller band was observed following infection with Ad-SIRT3SF (Fig 1B). The cellular distribution of the proteins in NIT1 cells was investigated with immunocytochemistry confocal analysis. SIRT3LF was localized in the mitochondria, while SIRT3SF was present mainly in the nucleus and partially in the cytoplasm (Fig 1C), consistent with the hypothesis that SIRT3SF could not be imported to the mitochondria since the import signal had been deleted. To confirm cellular localization of SIRT3LF and SIRT3SF, biochemical fractionation was also performed after infection of Ad-SIRT3LF and Ad-SIRT3SF into NIT1 cells. The processed SIRT3LF was abundantly detected in the mitochondria fraction and also detected in the nucleus fraction, suggesting that SIRT3LF was mainly localized in the mitochondria but also partially localized in the nucleus although nuclear localization was not detected in the immunocytochemistry analysis. Consistent with the immunocytochemistry result, SIRT3SF was detected in the nucleus and cytoplasm fraction (Fig 1D). Lower protein levels of SIRT3 were detected after Ad-SIRT3SF infection than after Ad-SIRT3LF infection, even though the transcription levels were similar (data not shown). Similar results were obtained when expression vectors for these two SIRT3 forms were transfected into COS7 cells (data not shown), leading us to speculate that the two forms have different stability. To test their stability, the protein-synthesis inhibitor cycloheximide was applied after transfection of SIRT3LF or SIRT3SF expression vectors into COS7 cells. While the level of SIRT3SF was rapidly reduced, the SIRT3 small form generated from SIRT3LF was quite stable, suggesting that SIRT3 is more stable in the mitochondria than in the nucleus (Fig 1E). To confirm that the SIRT3LF and SIRT3SF overexpressed by viral system are active in NIT1 cells, acetylated proteins were detected using Western blot analysis. After infection of Ad-SIRT3LF or Ad-SIRT3SF, acetylated proteins were dramatically reduced, indicating that overexpressed SIRT3LF and SIRT3SF are active (Fig 1F).

### Overexpression of SIRT3 ameliorates palmitate-induced cell death

**Fig 2:**
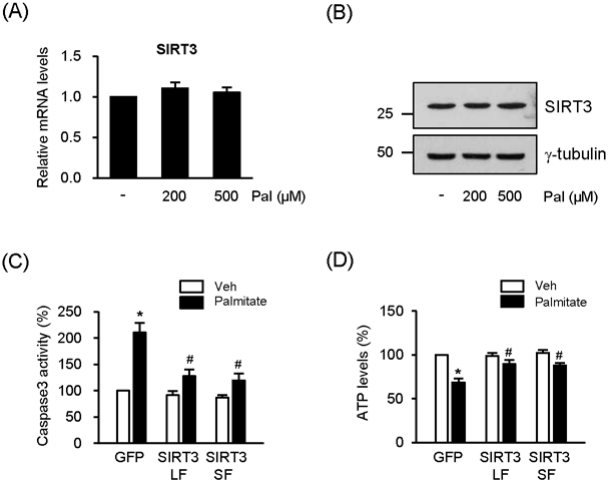
SIRT3 overexpression ameliorates reduction of viability by palmitate. (A) NIT1 cells were incubated with palmitate (Pal) as indicated. After 24 h incubation, SIRT3 mRNA levels were measured by real-time PCR. The mRNA level of untreated cells was expressed 1.0 and the others were as its relative values. Data are the means ± SEM of 5 independent experiments. (B) NIT1 cells were incubated with palmitate for 24h. Cell lysates (50 μg) were loaded on each lane of SDS-PAGE gels and immunoblotted with an anti-SIRT3 antibody. (C) NIT1 cells were infected with Ad-GFP, Ad-SIRT3LF, or Ad-SIRT3SF (all 50 MOI) and treated with palmitate (500 μM) for 24 h. Caspase 3 activity was measured [n = 6]. (D) NIT1 cells were infected with Ad-GFP, Ad-SIRT3LF, or Ad-SIRT3SF (all 50 MOI) and treated with palmitate (500 μM) for 8 h. Cellular ATP levels were determined [n = 4]. Caspase activity and ATP levels of the cells were expressed as percentage of values measured in the cells infected with Ad-GFP without palmitate. Data represent means ± SEM; *P < 0.05 for comparison with Ad-GFP infected cells without palmitate treatment; #*P* < 0.05 for the comparison with Ad-GFP infected cells treated with palmitate.

We tested whether SIRT3 expression is affected by palmitate treatment in NIT1 cells. The mRNA levels of SIRT3 were unchanged in response to palmitate, suggesting that palmitate did not affect the expression of SIRT3 (Fig 2A). Similarly, the protein levels of SIRT3 from the whole cell lysates were not changed by palmitate (Fig 2B). To test whether SIRT3 can improve viability of pancreatic β cells in lipotoxic conditions, NIT1 cells were infected with Ad-SIRT3LF or Ad-SIRT3SF and then treated with palmitate (500 μM) for 24 h. Palmitate treatment significantly increased caspase 3 activity in the cells infected Ad-GFP (control cells). However, the caspase 3 activity was decreased in the cells infected with Ad-SIRT3LF or Ad-SIRT3SF in the presence of palmitate (Fig 2C). ATP levels in the cells infected with Ad-SIRT3LF or Ad-SIRT3SF were much higher than those in the cells infected with Ad-GFP after palmitate treatment (Fig 2D). These results suggest that both SIRT3LF and SIRT3SF partially protect pancreatic β cells against palmitate-induced cell death.

### Overexpression of SIRT3 partially recovers glucose-stimulated insulin secretion

**Fig 3:**
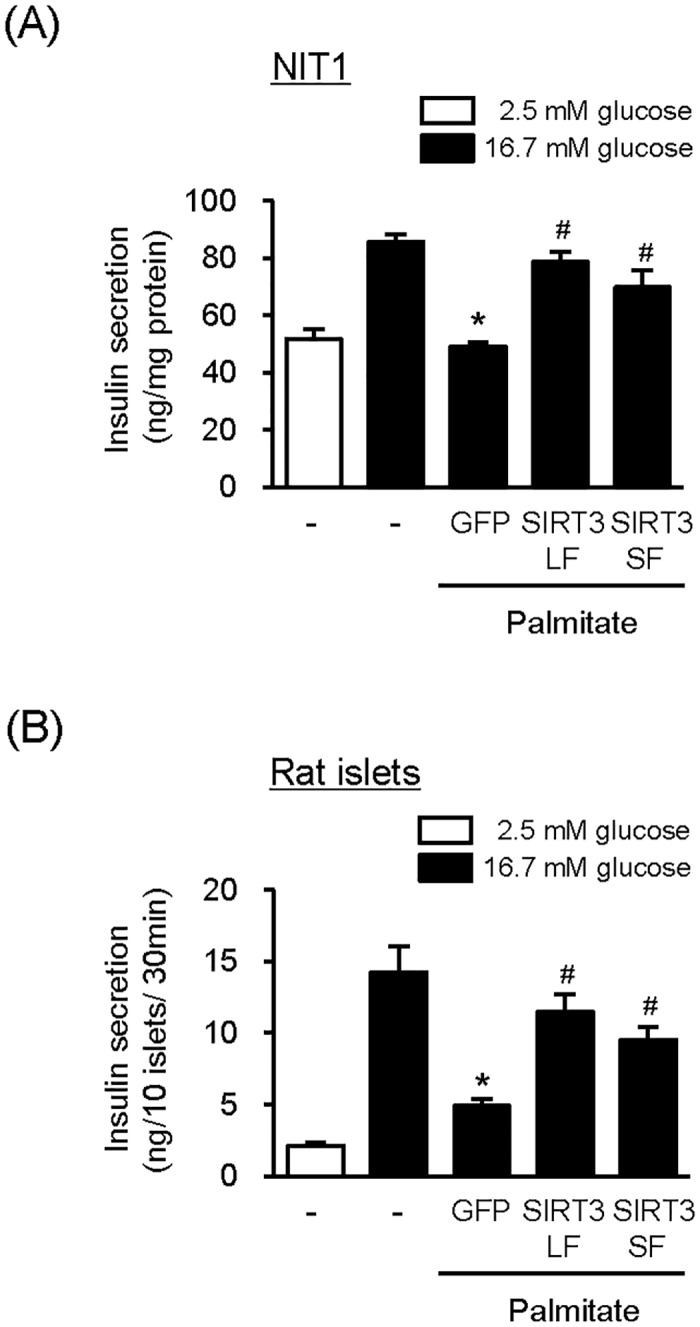
SIRT3 overexpression partially recovers GSIS in the presence of palmitate. (A) NIT1 cells were infected with Ad-GFP, Ad-SIRT3LF, or Ad-SIRT3SF (50 MOI) for 24 h and then treated with palmitate (200 μM) for additional 48 h. Secreted insulin was measured after incubation with low glucose (2.5 mM) or high glucose (16.7 mM) for 1 h. Data represent as means ± SEM [n = 6]; *P < 0.05 for the comparison with the value of high glucose treated cells without virus infection or palmitate treatment; #*P* < 0.05 for the comparison with the value of cells infected with Ad-GFP and treated with palmitate. (B) Pancreatic islets isolated from rats were infected with Ad-GFP, Ad-SIRT3LF, or Ad-SIRT3SF (100 MOI) for 24h and incubated with palmitate (400 μM) for additional 48 h. Secreted insulin from 10 islets was measured 30 min after each glucose exposure. Data represent as means ± SEM [n = 3]; *P < 0.05 for the comparison with the value of high glucose treated cells without virus infection or palmitate treatment; #*P* < 0.05 for the comparison with the value of cells infected with Ad-GFP and treated with palmitate.

When NIT1 cells were treated with 200 μM or lower concentrations of palmitate for 48 h, cell viability was not reduced (data not shown). Therefore, a lower concentration of palmitate (200 μM) was used in the experiment measuring glucose stimulated insulin secretion (GSIS) in order to induce β-cell dysfunction without cell death (Fig 3A). As predicted, a high concentration of glucose increased insulin secretion by 1.7-fold, which was completely abolished by palmitate treatment. However, when SIRT3LF or SIRT3SF was overexpressed in these cells, insulin secretion was enhanced responding to high concentration of glucose in the presence of palmitate (Fig 3A). In the same experimental condition, insulin protein levels in NIT1 cells were not changed in response to palmitate or overexpression of SIRT3, suggesting that the difference in the insulin secretion was not due to the levels of insulin expression (data not shown). To explain physiological relevance, we also tested whether SIRT3 overexpression could attenuate palmitate-induced GSIS impairment in rodent isolated islets. Insulin secretion was dramatically increased responding to high concentration of glucose in the isolated islets, and overexpression of SIRT3LF or SIRT3SF significantly enhanced insulin secretion in the presence of palmitate (Fig 3B). Based on the results, we conclude that overexpression of SIRT3LF or SIRT3SF ameliorates palmitate-induced β -cell dysfunction.

### Overexpression of SIRT3 represses palmitate-induced MAPK signaling and ER stress

**Fig 4:**
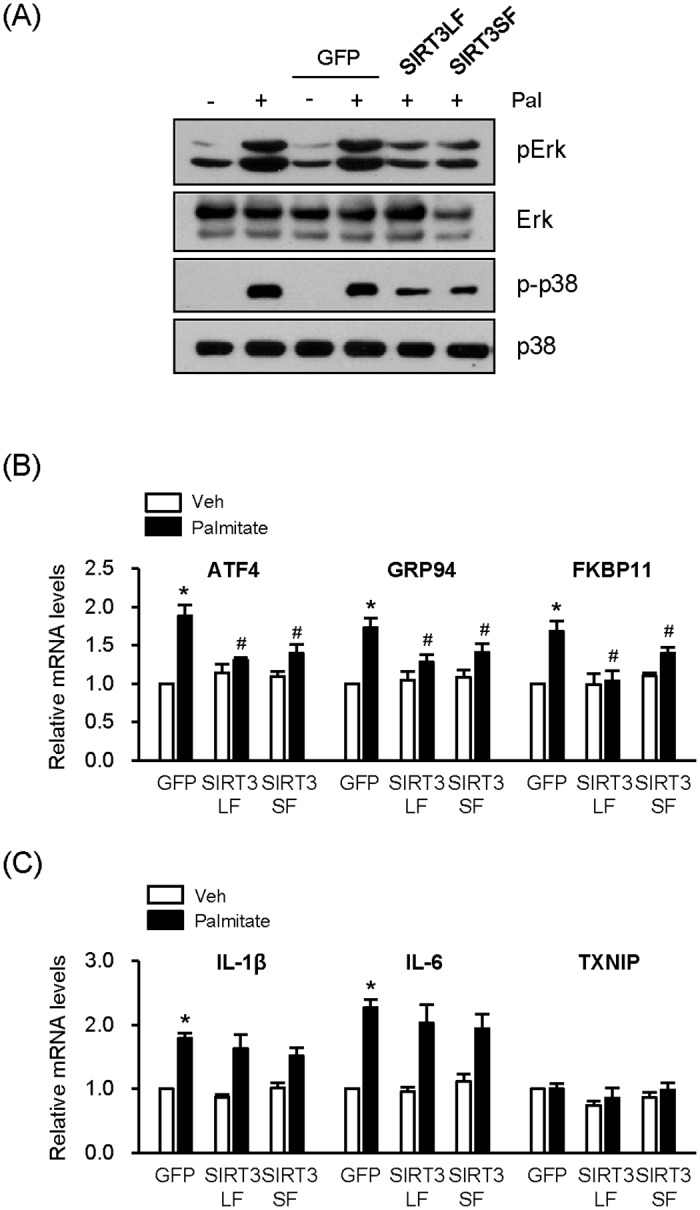
SIRT3 overexpression inhibits palmitate-induced MAPK activation and ER stress. (A) NIT1 cells were infected with Ad-GFP, Ad-SIRT3LF, or Ad-SIRT3SF (50 MOI) and treated with palmitate (500 μM) for 8 h. The cell lysates were subjected to Western blot analysis. (B and C) NIT1 cells were infected with Ad-GFP, Ad-SIRT3LF, or Ad-SIRT3SF (50 MOI) and treated with palmitate (500 μM) for 24 h. The mRNA levels of ATF4, GRP94, and FKBP11 [n = 6] (B) and the mRNA levels of IL-1β, IL-6 and TXNIP [n = 5] (C) were measured using real-time PCR. The mRNA levels of the cells infected with Ad-GFP without palmitate treatment was set to 1.0 and the others were expressed relative to that value; *P < 0.05 for the comparison with the value of the Ad-GFP-infected cells without palmitate; #*P* < 0.05 for the comparison with the value of Ad-GFP infected cells in the presence of palmitate.

When NIT1 cells were incubated with palmitate, the stress-induced MAP kinase signaling pathway was activated, evidenced by palmitate-induced phosphorylation of Erk or p38 (Fig 4A). Overexpression of SIRT3LF or SIRT3 SF reduced the phosphorylation of these MAPKs (Fig 4A). It is well known that palmitate induces ER stress in β cells, leading to accelerated expression of several ER stress-response genes including activating transcription factor 4 (ATF4), heat shock protein 90 beta 1 (HSP90b1, GRP94), and FK506 binding protein 11 (FKBP11) [19]. We measured mRNA levels of these genes by real-time PCR after palmitate treatment. The mRNA levels of ATF4, GRP94, and FKBP11 in palmitate-treated cells increased 1.7–1.9 fold over the untreated cells. However, the mRNA levels of these genes in palmitate-treated cells following infection of Ad-SIRT3LF were not different from untreated, control cells (Fig 4B). SIRT3SF also suppressed the palmitate-induced expression of the ER stress-related genes, but less effectively than SIRT3LF. These results demonstrate that SIRT3 suppresses palmitate-induced ER stress. We also tested whether SIRT3 overexpression affects palmitate-induced expression of proinflammatory cytokines such as IL1β and IL6. Palmitate-induced increases of IL1β and IL6 mRNA levels were still detected after the infection of Ad-SIRT3LF or Ad-SIRT3SF (Fig 4C). Either palmitate or SIRT3 did not affect the expression of an endogenous inhibitor of the antioxidant thioredoxin (TXNIP) which is known to mediate ER stress induced-activation of inflammation. Therefore, the results suggest that palmitate-induced inflammation is not mediated by ER stress and SIRT3 cannot regulate non ER stress-induced inflammation.

## Discussion

In this study, we determined the localization of overexpressed SIRT3 in NIT1 cells and investigated the effect of SIRT3 overexpression on palmitate-induced pancreatic β-cell dysfunction. According to our immunocytochemistry data, overexpressed full-length SIRT3 (SIRT3LF) was preferentially localized in mitochondria and its N-terminal truncated form (SIRT3SF) was mainly localized in the nucleus but was also detected in the cytoplasm. However, subcellular fractionation data showed that SIRT3LF was also detected in the nucleus. Therefore, it seemed that overexpressed SIRT3LF was partially localized in the nucleus although it was hardly detected by immunocytochemistry due to much smaller amount compared to the mitochondrial SIRT3. This result was consistent with the previous report demonstrating nuclear localization of SIRT3 [24].

Both SIRT3LF and SIRT3SF overexpression ameliorated palmitate-induced β-cell dysfunction and apoptosis. Due to the different cellular localization, the two forms may affect β-cell function by different mechanisms. However, it is also possible that nuclear SIRT3, generated from SIRT3LF and SIRT3SF, is more important for the effect on palmitate-induced β-cell dysfunction. Anyhow, it is clear that both SIRT3 forms alleviate palmitate-induced β-cell dysfunction via suppression of ER stress induction. Recent several studies have demonstrated that over-nutrition and insulin resistance increase the production of proinflammatory cytokines such as IL1β and IL6, which causes pancreatic β-cell dysfunction [26]. In addition, it has been reported that ER stress increases the production of IL1β through TXNIP up-regulation, which mediates ER stress-induced β cell death [27]. Our results showed that palmitate could not increase the expression of TXNIP, indicating that palmitate-induced inflammation was not mainly mediated by ER stress. This result is consistent with the previous report [28]. In addition, although ER stress responsive gene expression was reduced by SIRT3, expression of proinflammatory genes, which might not be mediated ER stress, was not affected, suggesting that SIRT3 suppresses ER stress induction but may not affect inflammation induction itself.

Exogenously expressed SIRT3 was easily detected after transfection of the SIRT3LF expression vector; however, there was less SIRTSF after transfection of the SIRTSF expression vector. The mitochondrial active short fragment of SIRT3, which resulted from translocation of SIRT3LF to the mitochondria and subsequent cleavage, was more stable than either the cytoplasmic or nuclear SIRT3SF (Fig 1E). This may account for the detection of SIRT3 mainly in the mitochondria, even though several workers have demonstrated that it is also detected in the cytoplasm and nucleus. Our data clearly show that SIRT3 in the nucleus or cytoplasm was active and effective in the protection of β cells from palmitate-induced dysfunction, despite being less abundant than mitochondrial SIRT3.

While it has been reported that SIRT1 mRNA level was down-regulated by palmitate in β cells [22], the level of SIRT3 mRNA was not affected by palmitate in our study, suggesting that expression of these two related proteins is differently regulated. SIRT3 mRNA levels have been reported to be reduced by pro-inflammatory cytokines such as TNFα and IL1β in β cells. In addition, SIRT3 mRNA levels are also reduced in the islets of diabetic patients [11]. Therefore, the evidence presented here on the effects of SIRT3 overexpression in pancreatic β cells may contribute to new and effective therapies for the treatment of diabetes.
